# The Passot Technique Revisited: No Vertical Scar Reduction Mammoplasty in Unmarried Females: A Case Series

**DOI:** 10.29252/wjps.10.3.84

**Published:** 2021-09

**Authors:** Debarati Chattopadhyay, Akshay Kapoor, Souradip Gupta, Nikhilesh Gaur, Sandipan Gupta

**Affiliations:** 1 Department of Burns and Plastic Surgery, All India Institute of Medical Sciences, Rishikesh, India; 2 Department of Plastic Surgery, Calcutta Medical College, West Bengal University of Health Sciences, Kolkata, India

**Keywords:** Reduction mammoplasty, Passot, Unmarried females

## Abstract

**BACKGROUND:**

Macromastia in adolescent girls is a distressing condition. There is an increase in the number of patients opting for reduction mammoplasty in the late teens. The semicircular horizontal method of breast reduction, first described by Passot in 1925 has the advantage of being able to do larger reduction, particularly suitable for pendulous breasts and having a hidden scar in the inframammary fold.

**METHODS:**

Eleven patients of adolescent macromastia were included in this study. It was conducted over a period of 4 years (2013-17) at two teaching institutions in Kolkata and Rishikesh, India. The mean age of the patients was 19.2 years. The Passot technique of reduction mammoplasty was performed in each case and the volume of resected breast tissue recorded by weighing the specimen. The aesthetic outcome was assessed by Lowery scale (volume, contour, placement of the breast mound and inframammary fold). Patient satisfaction was assessed after 6 months of follow up on a scale of 1 to 10, where 1-4 was poor, 5-6 fair, 7-8 good and 9-10 excellent.

**RESULTS:**

Mean total reduction per breast was 856 gm. Patients reported a mean decrease of cup size by 1.5. The aesthetic outcome was excellent in 6 patients and good in 5 patients. Patient satisfaction was excellent in 9 patients and good in 2 patients.

**CONCLUSION:**

Passot technique is a safe and effective technique of reduction mammoplasty and is especially useful in adolescent macromastia where the absence of visible scar on the breasts is very satisfying for the patients.

## INTRODUCTION

Macromastia in adolescent girls is a distressing condition because of the negative body image and psychological stress the girl endures. There is an increase in the number of patients opting for reduction mammoplasty in the late teens. In such cases, the choice of mammoplasty is a challenge to the reconstructive surgeons not only because of aesthetic reasons but also for the procedure’s future effect on nipple sensation, lactation, breast imaging and pregnancy-related breast changes^1^.

Macromastia in young patients usually results from virginal hypertrophy of the breast. Other causes may include obesity and rarely tumors^2^. 

The goals of reduction mammoplasty remain the same as described a century ago: To lift the breasts symmetrically to a youthful and natural form in proportion to other parts of the body with preservation of their function^3^. 

Reduction mammoplasty in unmarried females in our country possesses some unique problems. Besides the standard psychosocial and body image issues that are dealt with by the literature worldwide, the highest concern of our patients remains the scar. Plastic surgeons in India always come across these patients wanting no visible scar in their breasts for fear of social stigma ability to lactate comes as a secondary, albeit an important issue.

Thus many surgeons prefer the doughnut technique for reduction mammoplasty in unmarried females. The main limitation remains with the amount of reduction that is possible with this technique and it is not suitable for the larger pendulous breasts that we come across in our practice.

The Semicircular horizontal method of breast reduction, first described by Passot in 1925 was revived in its popularity by Lalonde et al in this millennium^4^^,^^5^. It has the advantage of being able to do larger reduction, particularly suitable for pendulous breasts and having a hidden scar in the inframammary fold.

The present study prospectively analyzed the results of Passot technique of reduction mammoplasty done in adolescent unmarried women in a teaching hospital in India. Eleven patients were studied with assessment of aesthetic outcomes, patient satisfaction with the results of surgery and symptomatic relief of macromastia.

*Aims of the Study*

1. To evaluate the aesthetic outcome in unmarried women undergoing reduction mammoplasty by Passot technique;

2. To evaluate the alleviation of symptoms of macromastia by this technique;

3. To evaluate the physical and psychosocial well-being of the patients after reduction mammoplasty by this technique.

## METHODS

Overall, 11 unmarried adolescent female patients were taken for the procedure at Calcutta Medical College, India and AIIMS Rishikesh, India from 2013 to 2017. 

An institutional ethics clearance for the study was taken from both centers. All procedures performed were in accordance with the ethical standards of the institutional and/or National Research Committee and with the 1964 Helsinki declaration and its later amendments or comparable ethical standards. The Institutional Ethics Clearance has been granted via letter-number AIIMS/IEC/16/44

After proper consent and photographic documentation, the markings were done with the patient in standing position.

**Fig 1:**
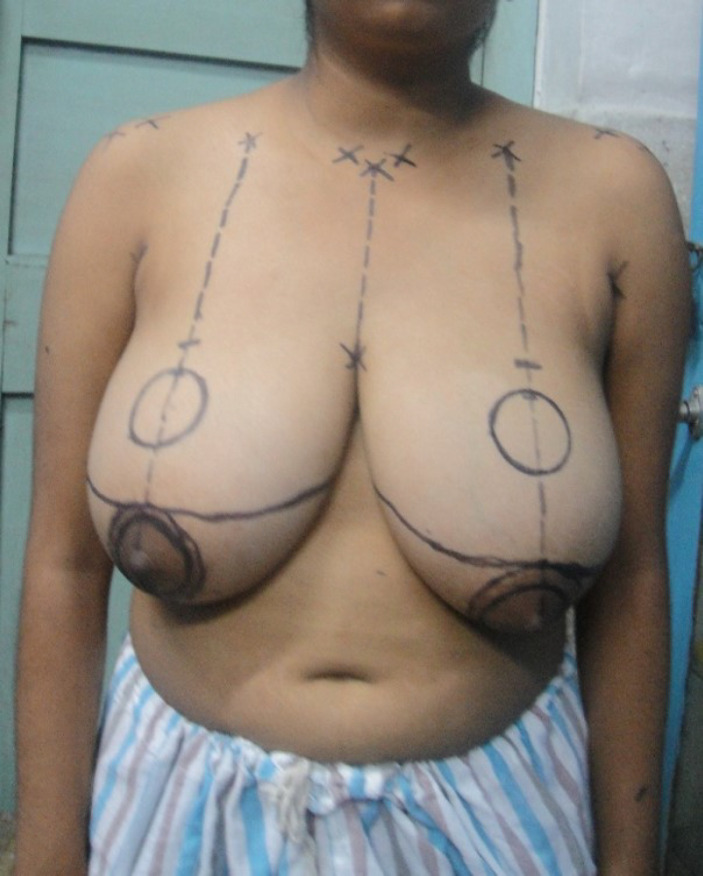
Marking for Passot technique of reduction mammoplasty

The positions of the future nipple-areola were determined following the standard wise pattern marking. The distance from the sternal notch to the nipple was kept at 19 cm-21 cm. The distance from the nipples to the inframammary fold was kept at 5cm-6 cm (Figure 1). To achieve a minimal areolar scar, the areola was measured at 4 cm-5 cm and the new areola site circle was drawn at 2.5–3.0 cm^6^.

The surgery was performed under general anesthesia with the patient in supine position. Tumescent solution was injected into the dermis of the inferior pedicle to minimize bleeding. The inferior dermoglandular pedicle thickness was kept at 2.5 cm and 6 cm-8 cm in width. It was not completely raised off the chest wall and a thick column of subareolar parenchyma was preserved^6^^.^ The upper skin flap was elevated between the subcutaneous tissue and Scarpa’s fascia and its thickness was about 1.5 cm^6^. The breast tissue was then removed as necessary.

At the new areola site, the circle of tissue marked was cut and the nipple-areola delivered through the hole. The areola was closed with interrupted absorbable dermal sutures. The inframammary fold incision was closed with buried dermal interrupted sutures and a running 3-0 absorbable suture. While closing, the skin was gathered in the central 1/3 of the incision line, thus maintaining the projection and shortening the scar^5^. Suction drains were placed in each breast.

The patients were followed up monthly for 2 months and examined clinically. At one year of follow-up, photographs were taken and the patients were given a subset of the Breast Q postoperative Version 2 questionnaire. They were asked about the following:

a. Satisfaction with breasts

b. Satisfaction with Nipples

c. Psychosocial well being

d. Physical well being

e. Satisfaction with outcome

## RESULTS

Mean age of the patients was 19.6 yr (Range 16 to 23.5 yr). Mean total reduction per breast was 856 gm (Range 450 gm to 1130 gm). Preoperatively, the mean distance from the sternal notch to the nipple was 32.8 cm (Range 28 cm to 37.9 cm). Postoperatively, the mean distance from the sternal notch to the nipple was 20.9 cm (range, 19.8 to 23 cm) (Table 1). Preoperatively, the median bra size was a 38 DD cup. Postoperatively, the median bra size was 34 C cup. Patients reported a mean decrease in bra cup size of 1.5 cup sizes (Range 1 to 3). The overall operative morbidity was minimal with only two minor complications (Table 1). 

The mean follow-up was 16.1 months (range: 12−18.2 months). The aesthetic and psychosocial outcomes were assessed at one year follow up with the” Breast Q Postoperative version 2”(Table 2). Median score of satisfaction with the breasts was 78, Satisfaction with Nipples was 18, Psychosocial outcomes was 88, physical wellbeing was 90 and satisfaction with the surgery was 86. That means that almost all the patients had excellent symptomatic relief with very good aesthetic outcomes (Figure 2 a,b,c and Figure 3a, b).

## DISCUSSION

Reduction mammoplasty in unmarried females in our country possess unique problems for the plastic surgeons because of the concerns over scarring and the requirement of an unoperated look as well as the probability of lactation in the future. A hidden scar seems to be the foremost concern of these patients requiring reduction mammoplasty. A surgeon here often has to offer the periareolar technique though it might not be suitable for the larger breasts, just because the patients do not want any scar over the visible part of the breast as breast surgeries in the more conservative society is a deterrant for marriage for the girl.

The semicircular horizontal technique of breast reduction, first described by Passot^4^ seems to be uniquely suitable for reduction mammoplasties of unmarried females in our country as it does away with the vertical scar over the breast. We followed the modifications by Lalonde et al and kept a 2.5 cm thick inferior pedicle as well as a 1.5 cm thick skin flap for the safety of vascular supply.

The advantages of this technique lies in its hidden periareolar and inframammary scar. As Lalonde et al had proposed the scars lie in the part of the breast which is hidden when the woman looks in the mirror^5^. Moreover, the technique is very suitable for large pendulous breasts. In our study, successful reduction could be done with the mean distance from sternal notch to the areola being 32.8 cm. The result reflects that of a previous study where this technique was used^6^.

The main criticism of this technique has been that it produces a more boxy appearance of the breast. Improvement of projection is usually managed by breast shaping sutures^5^. We did not use such sutures. Instead we kept did the following manoeuvres to improve projection.

1) The width of the dermoglandular pedicle was kept at 6-8 cm. A wider pedicle causes difficulty during skin closure.

2) The most important step for maintain the projection is the closure of the upper skin flap, which when closed uniformly causes the flatness of the breasts. In our study, the skin excess was bunched up in the middle 1/3^rd^ of the skin incision to maintain the breast projection. The resultant creases smoothened out in a few months^7^. 

There has been numerous controversies in the past of which is the best technique for reduction mammoplasty to preserve the maximum potency of lactation. Traditionally the inferior pedicle was thought to be the best in this regard. After extensive research, there was no significant association between superiorly, medially, or inferiorly-based reduction mammoplasties and lactational performance^8^. Recently, whichever technique that preserves an intact column of subareolar parenchyma has a better chance of fruitful lactation^8^. In our technique utilized the inferior dermoglandular pedicle and did not raise it completely from the chest wall, thus preserving a significant amount of the subareolar parenchyma. Two of our patients have been able to lactate till date.

Most of our patients reported a good nipple sensation postoperatively. This result again reflects similar results from a previous study^6^. The elevation of the thick upper skin between the subcutaneous tissue and Scarpa’s fascia seems to contribute to this.

Overall, the procedure described herein is suitable for reducing mammoplasty and specifically for reduction in unmarried females in our country. Its advantages are as follows: it achieves an excellent amount of reduction, gives a pleasing aesthetic appearance with sensate nipple and retains good chance of lactation.

**Fig 2a:**
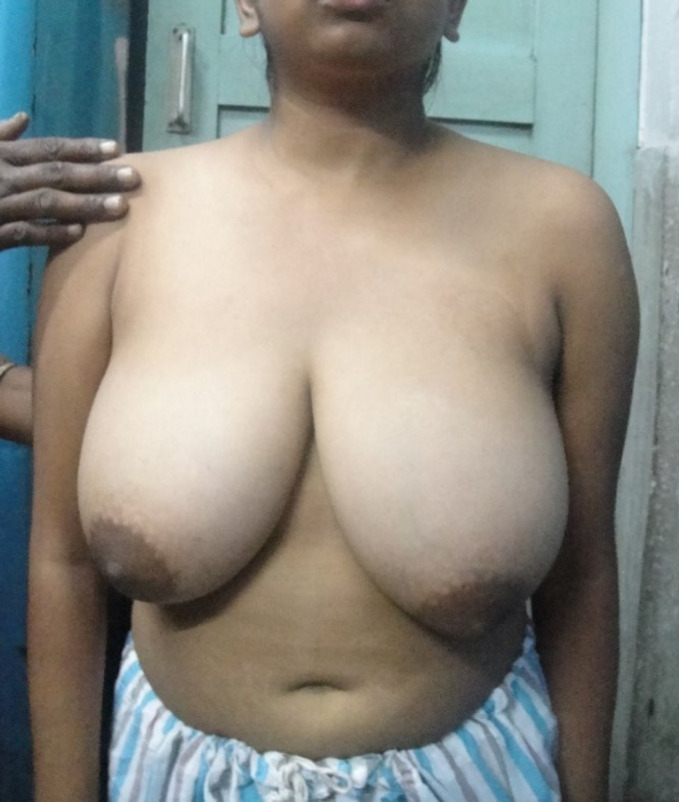
Preoperative picture of Patient 1

**Fig 2b:**
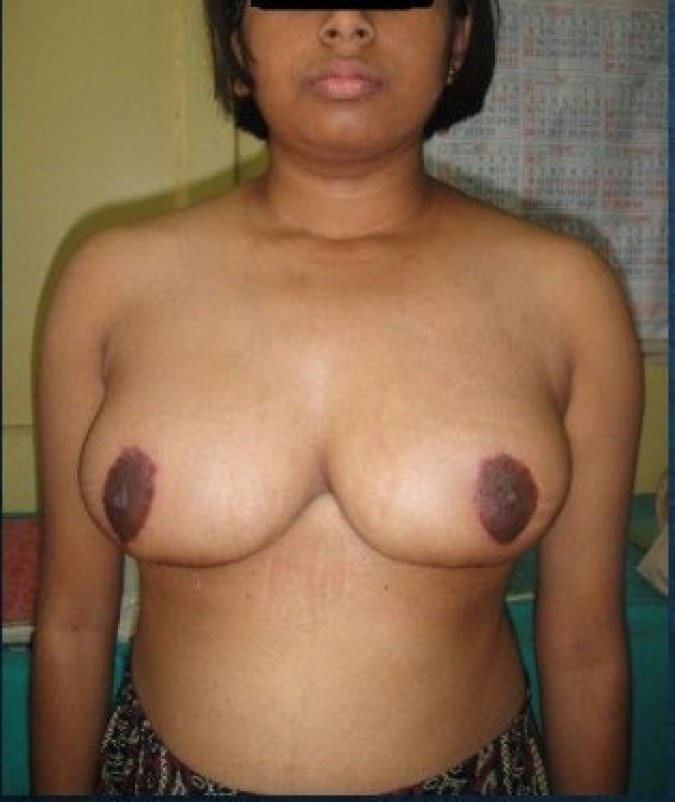
1 year follow up of Patient 1(Front view)

**Fig. 3a:**
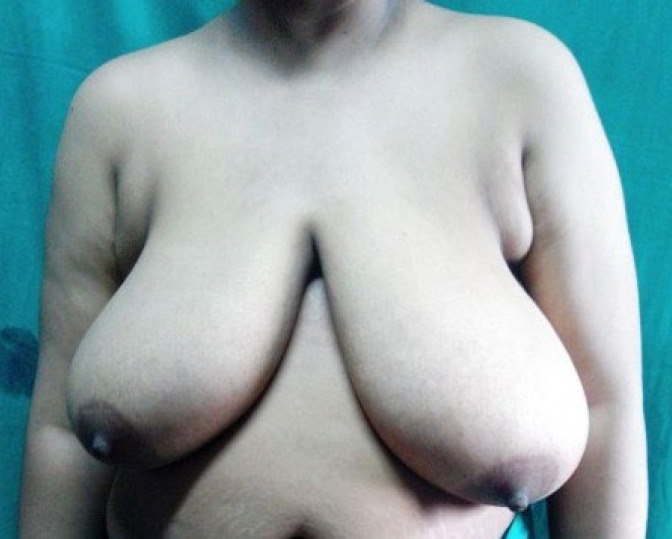
Preoperative picture of Patient 2

**Fig. 3b:**
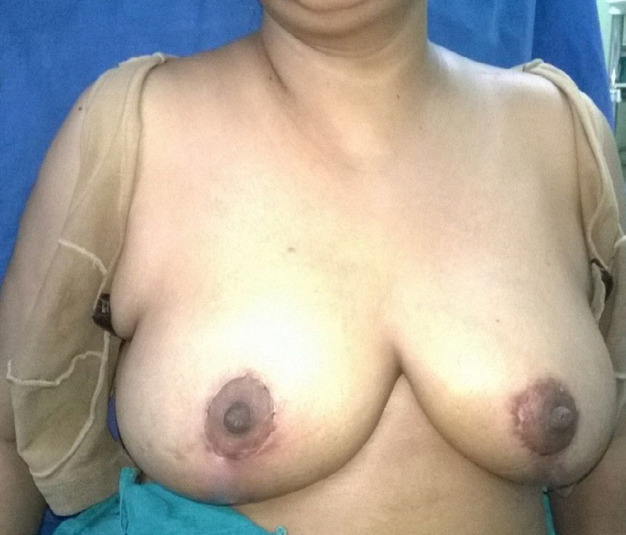
1 year follow up of Patient 2 (Front view)

**Table 1:** Patient details and reduction characteristics

Serial no patients	Age in years	SN to nipple distance in cm preop	SN to nipple distance in cm postop	Cup size preop	Cup size Post op	Reduction volume in grams (Mean)	Complications
1	16	29.1	20	36DD	34C	880	
2	20.3	37.9	22.1	38DD	36C	1130	Seroma
3	23.5	31.4	19.8	36DD	34C	670	
4	17.9	35.5	20.2	38DD	34C	950	
5	18.6	29.9	19.8	38D	34C	930	Seroma
6	22.1	28	19.9	36DD	34C	450	
7	18.9	36.3	21.5	38DD	34C	1100	
8	15.8	36	23	38DD	34D	850	
9	19.6	32	22	38DD	34C	740	
10	21.2	30.8	21.8	38DD	36B	690	
11	21.7	33.5	20	36DD	34C	1020	

**Table 2:** Results of the Breast Q questionnaire

Serial number of patients	Satisfaction with breasts (max score=100)	Satisfaction with nipples (max score=20)	Psychosocial well being (max score =100)	Physical well being (max score=100)	Satisfaction with outcome (max score=100)
1	82	18	88	90	86
2	78	20	93	90	86
3	92	18	84	90	86
4	78	18	88	100	100
5	78	18	84	90	86
6	78	18	88	100	100
7	78	17	88	90	86
8	78	20	93	90	100
9	86	18	88	90	100
10	78	17	88	90	86
11	78	18	88	1001	86

## CONCLUSION

The Passot technique of reduction mammoplasty achieve the main goal of successful breast reduction which is creating a functionally and aesthetically pleasing breast. Moreover, this technique seems to be uniquely suitable for reduction of macromastia of unmarried females because it satisfies the primary determining factors for the technique selection in such patients: scar and lactation. Thus this technique can be utilized in the armamentarium of reduction mammoplasty in adolescent females to alleviate the increased social, psychological, and physical strain caused by macromastia.

## DISCLOSURE OF INTEREST

The authors declare that they have no conflicts of interest concerning this article.

## FUNDING

The authors declare that they have not received any financial support for this study and have no financial disclosures.
